# The Prognostic Significance of the Continuous Administration of Anti-PD-1 Antibody *via* Continuation or Rechallenge After the Occurrence of Immune-Related Adverse Events

**DOI:** 10.3389/fonc.2021.704475

**Published:** 2021-09-24

**Authors:** Toshiya Fujisaki, Satoshi Watanabe, Takeshi Ota, Kohei Kushiro, Yusuke Sato, Miho Takahashi, Aya Ohtsubo, Satoshi Shoji, Koichiro Nozaki, Kosuke Ichikawa, Satoshi Hokari, Rie Kondo, Takao Miyabayashi, Tetsuya Abe, Satoru Miura, Hiroshi Tanaka, Masaaki Okajima, Masaki Terada, Naoya Matsumoto, Takashi Ishida, Akira Iwashima, Kazuhiro Sato, Hirohisa Yoshizawa, Nobumasa Aoki, Masachika Hayashi, Yasuyoshi Ohshima, Toshiyuki Koya, Toshiaki Kikuchi

**Affiliations:** ^1^ Department of Respiratory Medicine and Infectious Diseases, Niigata University Graduate School of Medical and Dental Sciences, Niigata, Japan; ^2^ Department of Respiratory Medicine, Niigata Prefectural Shibata Hospital, Niigata, Japan; ^3^ Department of Respiratory Medicine and Infectious Diseases, Niigata City General Hospital, Niigata, Japan; ^4^ Department of Internal Medicine, Niigata Cancer Center Hospital, Niigata, Japan; ^5^ Department of Respiratory Medicine, Saiseikai Niigata Hospital, Niigata, Japan; ^6^ Department of Respiratory Medicine, Nishi-Niigata Chuo National Hospital, Niigata, Japan; ^7^ Department of Respiratory Medicine, Niigata Prefectural Central Hospital, Joetsu, Japan; ^8^ Department of Respiratory Medicine, Nagaoka Chuo General Hospital, Nagaoka, Japan; ^9^ Department of Respiratory Medicine, Nagaoka Red Cross Hospital, Nagaoka, Japan; ^10^ Department of Respiratory Medicine, Niigata Medical Center, Niigata, Japan

**Keywords:** drug therapy, immune-related adverse event, immunology, NSCLC, PD-1

## Abstract

**Objectives:**

Although immune checkpoint inhibitors (ICIs) have been shown to improve overall survival (OS) in advanced non-small-cell lung cancer (NSCLC) patients, ICIs sometimes cause various types of immune-related adverse events (irAEs), which lead to the interruption of ICI treatment. This study aims to evaluate the clinical significance of the continuation of ICIs in NSCLC patients with irAEs and to assess the safety and efficacy of the readministration of ICIs after their discontinuation due to irAEs.

**Methods:**

We retrospectively identified patients with advanced NSCLC who were treated with first- to third-line anti-programmed cell death-1 (PD-1) therapy from January 2016 through October 2017 at multiple institutions belonging to the Niigata Lung Cancer Treatment Group. Progression-free survival (PFS) and OS from the initiation of ICI treatment were analyzed in patients with and without irAEs, with and without ICI interruption, and with and without ICI readministration. A 6-week landmark analysis of PFS and OS was performed to minimize the lead-time bias associated with time-dependent factors.

**Results:**

Of 231 patients who received anti-PD-1 antibodies, 93 patients (40%) developed irAEs. Of 84 eligible patients with irAEs, 32 patients (14%) continued ICIs, and OS was significantly longer in patients who continued ICIs than that in patients who discontinued ICIs [not reached (95% CI: NE-NE) *vs*. not reached (95% CI: 22.4–NE); p = 0.025]. Of 52 patients who discontinued ICIs, 14 patients (6.1%) readministered ICIs, and OS in patients with ICI readministration was significantly longer than that in patients without ICI readministration [not reached (95% CI: NE-NE) *vs*. not reached (95% CI: 8.4–NE); p = 0.031].

**Conclusion:**

The current study demonstrated that both the continuation and readministration of ICIs after irAE occurrence improved OS compared to the permanent interruption of ICIs in NSCLC patients with ICI-related irAEs.

## Introduction

Immune checkpoint inhibitors (ICIs), such as anti-programmed cell death-1 (PD-1) and anti-programmed cell death ligand-1 (PD-L1) antibodies, have achieved durable responses in some patients with non-small-cell lung cancer (NSCLC) (1–6). Anti-PD-1/PD-L1 therapy has become the standard of care for advanced NSCLC patients.

Treatment with ICIs is often accompanied by immune-related adverse events (irAEs), and irAEs can be lethal or the main reason for the discontinuation of ICIs. The decision of whether to continue or discontinue ICIs after the occurrence of irAEs is generally based on the type of irAE and its severity (7). We previously reported that ICI-related interstitial lung disease (ILD), whose appearance was ground-glass opacities (GGOs), was a significant predictor of poor survival outcomes (8). However, recent evidence has demonstrated that the occurrence of irAEs is associated with better survival outcomes in patients with NSCLC (9–13). Several retrospective studies have also shown that there were some cases in which the effects of ICIs were sustained even after the discontinuation of treatment due to ICI-related irAEs (14, 15). In contrast, a retrospective study reported that the interruption of ICIs due to irAEs was associated with a lower overall survival (OS) than continuous ICI treatment (16). Furthermore, the clinical outcomes of the rechallenge of ICIs in patients who recovered from irAEs remain unclear.

This study aims to assess the significance of the continuation of ICIs in NSCLC patients who developed irAEs and to evaluate the safety and efficacy of the readministration of ICIs in patients who discontinued ICI treatment due to ICI-related irAEs.

## Materials and Methods

### Study Design and Patients

We retrospectively analyzed the medical records of patients with advanced NSCLC who were treated with single-agent anti-PD-1 as first- to third-line therapy at multiple institutions belonging to the Niigata Lung Cancer Treatment Group from January 2016 to October 2017. To prevent selection bias, all consecutive patients who met eligibility were enrolled. This study was approved by the institutional review board of each participating institution.

### Study Assessment

The following data were collected retrospectively for all patients: demographics, phenotypes of cancers, types of anti-PD-1 therapies, and irAEs. Treatment responses were evaluated according to the Response Evaluation Criteria in Solid Tumors (RECIST) criteria version 1.1. Each irAE was graded according to the Common Terminology Criteria for Adverse Events (CTCAE) version 4.0. Progression-free survival (PFS) was measured as the time from the start of anti-PD-1 therapy to progressive disease (PD) or death due to any cause. OS was measured as the time from the first administration of immunotherapy to death due to any cause. The objective response rate (ORR) was defined as the percentage of patients assessed as having complete response (CR) or partial response (PR) of all patients treated with anti-PD-1 therapy. The disease control rate (DCR) was defined as the percentage of patients assessed as having CR, PR, or stable disease (SD) of all patients treated with anti-PD-1 therapy. Treatment interruption was defined as either the delay or cessation of ICI treatment due to irAEs. Patients with ICI readministration were defined as those who were readministered a PD-1 inhibitor at least one time after the interruption of ICI treatment due to irAEs. Patients without ICI readministration were defined as those whose ICI treatment was permanently stopped due to irAEs.

### Statistical Analysis

**Figure 1:**
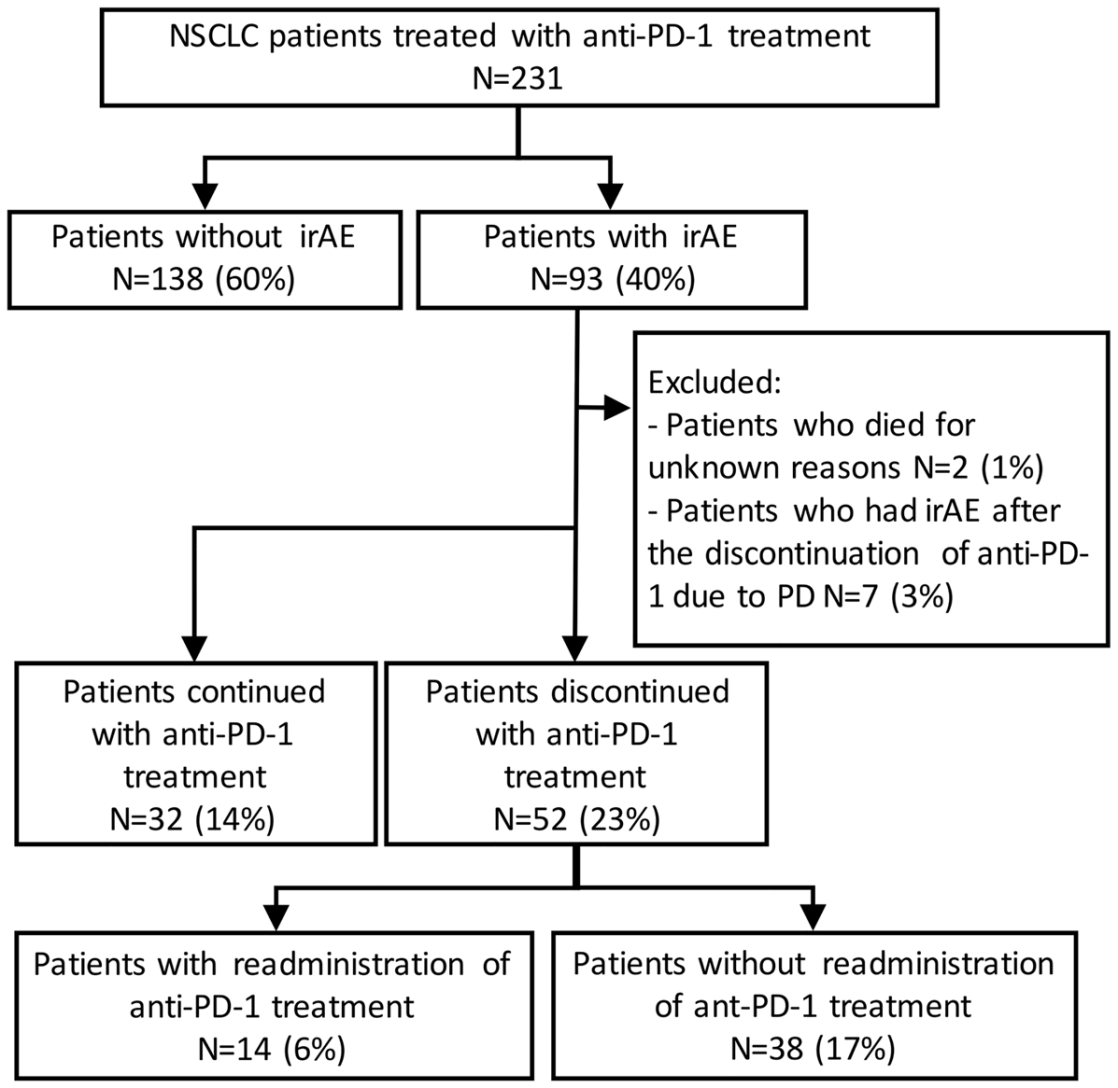
Patient flow diagram. NSCLC, non-small-cell lung cancer; PD-1, programmed cell death-1; irAE, immune-related adverse event.

**Figure 2:**
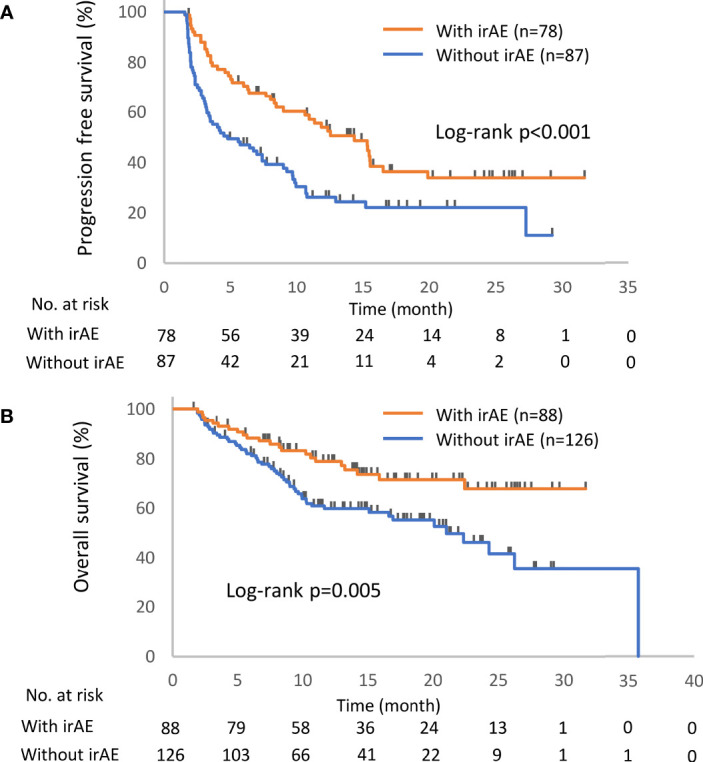
Kaplan–Meier curves for the 6-week landmark analysis of the progression-free survival **(A)** and overall survival **(B)** of patients with or without irAEs. IrAE, immune-related adverse event.

**Figure 3:**
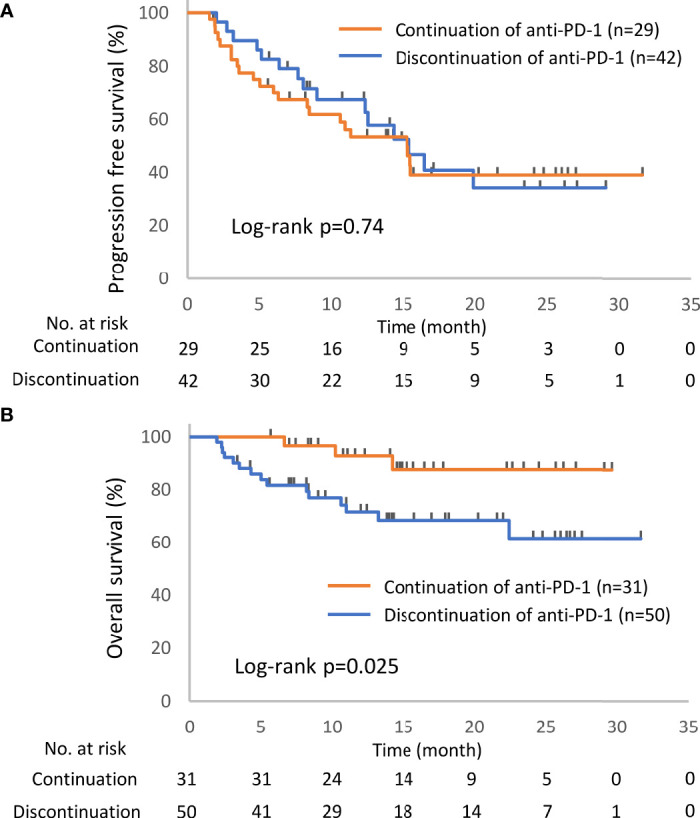
Kaplan–Meier curves for the 6-week landmark analysis of the progression-free survival **(A)** and overall survival **(B)** of patients continuing or stopping PD-1 treatment after irAE occurrence. PD-1, programmed cell death-1; irAE, immune-related adverse event.

**Figure 4:**
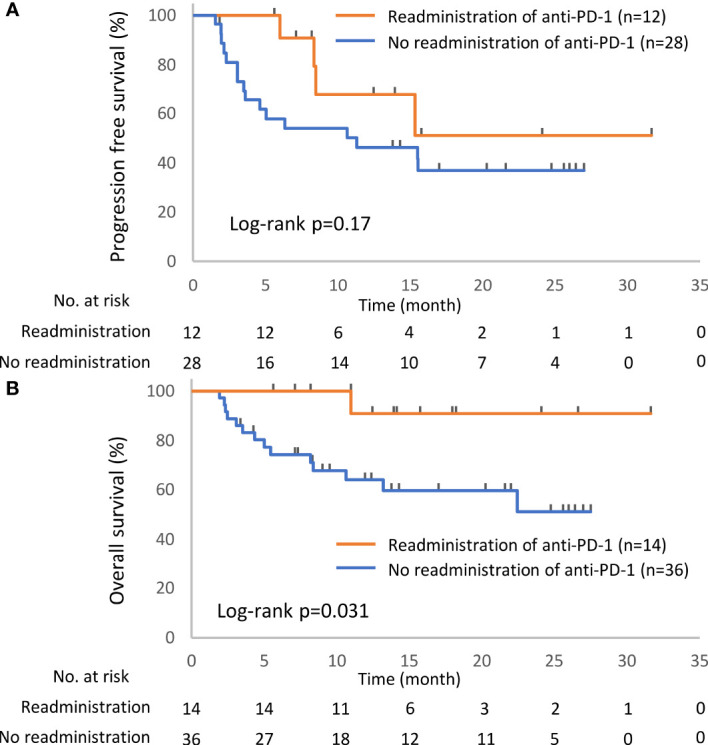
Kaplan–Meier curves for the 6-week landmark analysis of the progression-free survival **(A)** and overall survival **(B)** of patients with or without the readministration of anti-PD-1 treatment after the discontinuation of anti-PD-1 treatment due to irAEs. PD-1, programmed cell death-1; irAE, immune-related adverse event.

Kaplan–Meier survival curves were constructed for PFS and OS, and differences between groups were identified using the log-rank test. The univariate Cox proportional hazards model was used to assess the effects of the presence of irAEs, the continuation of ICIs, and the readministration of ICIs on PFS and OS. Continuous variables are presented as the median (range) and were compared by two-sided *t*-tests. Comparisons between groups were performed by Fisher’s exact test or the chi-square test. To minimize the lead-time bias associated with time-dependent factors, we performed a 6-week landmark analysis including only patients who were alive or whose disease was under control at 43 days after the initiation of anti-PD-1 therapy, which is the median time of onset of irAEs, for PFS and OS. For this 6-week landmark analysis, we excluded 66 patients for PFS and 17 patients for OS in the analysis of **Figure 2**, 13 patients for PFS and three patients for OS in the analysis of **Figure 3**, and 12 patients for PFS and two patients for OS in the analysis of **Figure 4** because these patients had PD or died for any cause within 6 weeks of initiation of anti-PD-1 therapy. Additionally, we excluded seven patients who experienced irAEs after the discontinuation of anti-PD-1 treatment due to PD and two patients who died suddenly for unknown reasons after developing irAEs (**Figures 1**, **3** and **Table 4**). All the reported p-values were two-sided, and p < 0.05 was considered significant. Statistical analysis was performed using JMP 14.2.0 statistical software (SAS Institute, Cary, NC, USA).

## Results

### Patient Characteristics

In total, 231 patients were enrolled in this study. Among these patients, 93 patients (40%) developed irAEs (**Figure 1**). The baseline characteristics at the initiation of anti-PD-1 therapy of patients with and without irAEs are presented in **Table 1**. The percentages of males, current or former smokers, squamous cell carcinoma, and pembrolizumab use were significantly higher in patients with irAEs than those in patients without irAEs. On the other hand, the percentage of epidermal growth factor receptor (*EGFR)* mutations was lower in patients with irAEs than that in patients without irAEs. Other clinical features, including age, Eastern Cooperative Oncology Group performance status, treatment line, and PD-L1 expression, were not significantly different.

**Table 1:** Patients’ characteristics at anti-PD-1 therapy.

Clinical feature		With irAEs (total, n = 93)	Without irAEs (total, n = 138)	p*-*value
Median age, years (range)		67 (41–84)	68 (38–82)	0.4^a^
Sex, n (%)	Female/male	14 (15)/79 (85)	41 (30)/97 (70)	0.016^b^
Smoking status, n (%)	Current or former	84 (90)	102 (74)	0.004^b^
	Never	9 (9.7)	36 (26)	
PS, n (%)	0–1	80 (86)	108 (78)	0.19^b^
	≥2	13 (14)	30 (22)	
Stage, n (%)	III	12 (13)	9 (7)	0.23^b^
	IV	45 (48)	76 (55)	
	Recurrent	36 (39)	53 (38)	
Histology, n (%)	Adenocarcinoma	36 (39)	96 (69)	<0.001^b^
	Squamous cell carcinoma	46 (49)	34 (25)	
	Others	11 (12)	8 (6)	
Driver mutation, n (%)	*EGFR*	1 (1)	12 (9)	0.017^c^
Treatment line of anti-PD-1 therapy, n (%)	first line	21 (23)	17 (12)	0.06^b^
	second, third line	72 (77)	121 (88)	
PD-L1 expression, n (%)	≥50%	28 (30)	26 (19)	0.16^c^
	1%–49%	5 (5)	8 (6)	
	<1%	7 (8)	7 (5)	
	Unknown	53 (57)	97 (70)	
Anti-PD-1 therapy, n (%)	Nivolumab	63 (68)	113 (82)	0.02^b^
	Pembrolizumab	30 (32)	25 (18)	
Median duration between initial anti-PD-1 treatment to the first irAE onset, days (range)		43 (0–522)		

Differences between groups were identified using ^a^Student’s t-test, ^b^chi-square test, or ^c^Fisher’s exact test. PS, performance status; PD-1, programmed cell death-1; PD-L1, programmed cell death ligand 1; irAE, immune-related adverse event; EGFR, epidermal growth factor receptor.

### Association of Immune-Related Adverse Events With Clinical Outcomes

The distribution of irAEs is shown in **Table 2**. The Kaplan–Meier curves of the 6-week landmark analysis for PFS and OS in patients with and without irAEs are shown in **Figure 2**. The median PFS was significantly longer in patients with irAEs than that in patients without irAEs [14.3 (95% CI: 9.0–16.5) *vs*. 4.8 (95% CI: 3.2–7.6); p < 0.001]. The median OS was also significantly longer in patients with irAEs than that in patients without irAEs [not achieved (95% CI: NE–NE) *vs*. 21.0 (95% CI: 15.1–NE); p = 0.005]. The hazard ratios estimated by the Cox proportional hazards model were as follows: the PFS hazard ratio was 0.51 (95% CI: 0.34–0.75; p < 0.001), and the OS hazard ratio was 0.49 (95% CI: 0.30–0.81; p = 0.005). Furthermore, the ORR and DCR were significantly higher in patients with irAEs than those in patients without irAEs (**Table 3**).

**Table 2:** Distribution of irAEs.

Phenotypes of irAEs	Total (n = 231)	CTCAE G3–4	CTCAE G5	Therapy continued	Systemic steroid	IrAEs improved
Pneumonitis, n (%)	33 (14)	11 (5)	1 (0.4)	1 (0.4)	24 (10)	31 (13)
Thyroid dysfunction, n (%)	26 (11)	2 (1)	0 (0)	17 (7.4)	0 (0)	23 (10)
Rash, n (%)	14 (6)	2 (1)	0 (0)	8 (3.5)	3 (1)	13 (6)
Pyrexia, n (%)	10 (4.3)	1 (0.4)	0 (0)	6 (3)	1 (0.4)	10 (4.3)
Diarrhea/colitis, n (%)	9 (4)	0 (0)	0 (0)	5 (2)	3 (1)	6 (3)
Adrenal insufficiency, n (%)	7 (3)	2 (1)	0 (0)	3 (1)	7 (3)	7 (3)
Infusion reaction, n (%)	6 (3)	0 (0)	0 (0)	3 (1)	3 (1)	6 (3)
Pruritus, n (%)	3 (1)	0 (0)	0 (0)	2 (1)	0 (0)	2 (1)
Liver dysfunction, n (%)	3 (1)	1 (0.4)	0 (0)	0 (0)	1 (0.4)	3 (1)
Anorexia, n (%)	2 (1)	0 (0)	0 (0)	0 (0)	0 (0)	1 (0.4)
Neuropathy, n (%)	2 (1)	1 (0.4)	0 (0)	0 (0)	2 (1)	1 (0.4)
Fatigue, n (%)	2 (1)	0 (0)	0 (0)	1 (0.4)	0 (0)	1 (0.4)
Nausea, n (%)	1 (0.4)	0 (0)	0 (0)	0 (0)	0 (0)	1 (0.4)
Proteinuria, n (%)	1 (0.4)	0 (0)	0 (0)	1 (0.4)	0 (0)	1 (0.4)
Uveitis, n (%)	1 (0.4)	0 (0)	0 (0)	0 (0)	0 (0)	1 (0.4)
Thrombocytopenia, n (%)	1 (0.4)	0 (0)	0 (0)	0 (0)	0 (0)	0 (0)
Autoimmune myositis, n (%)	1 (0.4)	1 (0.4)	0 (0)	0 (0)	1 (0.4)	0 (0)
Fulminant type 1 diabetes, n (%)	1 (0.4)	1 (0.4)	0 (0)	0 (0)	0 (0)	1 (0.4)
Depression, n (%)	1 (0.4)	1 (0.4)	0 (0)	1 (0.4)	0 (0)	1 (0.4)
Death NOS, n (%)	1 (0.4)	0 (0)	1 (0.4)	0 (0)	0 (0)	0 (0)

IrAE, immune-related adverse event; CTCAE, Common Terminology Criteria for Adverse Events; Death NOS, death that cannot be attributed to a CTCAE term associated with Grade 5.

**Table 3:** Association between irAEs and treatment responses.

	All (n = 231)	With irAE (n = 93)	Without irAE (n = 138)	p-value
CR, n (%)	5 (2)	4 (4)	1 (1)	
PR, n (%)	69 (30)	41 (44)	28 (20)	
SD, n (%)	51 (22)	22 (24)	29 (21)	
PD, n (%)	96 (42)	19 (20)	77 (56)	
NE, n (%)	10 (4)	7 (8)	3 (2)	
ORR, n (%)	74 (32)	45 (48)	29 (21)	<0.001^a^
DCR, n (%)	125 (54)	67 (72)	58 (42)	<0.001^a^

Differences between groups were identified using ^a^chi-square test. IrAE, immune-related adverse event; CR, complete response; PR, partial response; SD, stable disease; PD, progressive disease; NE, not evaluable; ORR, objective response rate; DCR, disease control rate

### Association of Immune Checkpoint Inhibitor Interruption Due to Immune-Related Adverse Events With Clinical Outcomes

Of the 93 patients with irAEs, 32 patients continued anti-PD-1 treatment, and 52 patients discontinued anti-PD-1 treatment (**Supplementary Table S1**). Patients who died for unknown reasons (n = 2) and those who had irAEs after the discontinuation of anti-PD-1 treatment due to PD (n = 7) were excluded from the following analysis (**Figure 1**). The Kaplan–Meier curves of the 6-week landmark analysis for patients who continued and discontinued anti-PD-1 therapy are shown in **Figure 3**. The median PFS was not different between patients who continued and discontinued anti-PD-1 treatment [15.4 (95% CI: 9.0–NE) *vs*. 15.3 (95% CI: 8.3–NE); p = 0.76]. However, the median OS was significantly longer in patients who continued ICIs than that in patients who discontinued ICIs [not reached (95% CI: NE–NE) *vs*. not reached (95% CI: 22.4–NE); p = 0.025]. The hazard ratios estimated by the Cox proportional hazards model were as follows: the PFS hazard ratio was 0.9 (95% CI: 0.47–1.74; p = 0.76), and the OS hazard ratio was 0.27 (95% CI: 0.077–0.92; p = 0.036). In terms of irAE phenotypes, the percentage of patients who experienced immune-related pneumonitis was higher in the anti-PD-1 treatment interruption group than that in the anti-PD-1 continuation group (**Table 4**). On the other hand, the percentage of patients who experienced immune-related thyroid dysfunction was higher in the anti-PD-1 continuation group than that in the anti-PD-1 interruption group. As expected, the percentage of patients who experienced grade 3 or higher irAEs was higher in the anti-PD-1 interruption group than that in the anti-PD-1 continuation group. Other phenotypes of irAEs, the timing of the first irAE, ORR, and DCR were not different among patients with anti-PD-1 interruption and those with anti-PD-1 continuation.

**Table 4:** Characteristics of the initial irAEs and clinical courses, related treatment interruption.

		Anti-PD-1 treatment interruption (n = 52)	Anti-PD-1 treatment continuation (n = 32)	p-value
Phenotypes of irAE	Pneumonitis, n (%)	29 (54)	1 (3)	<0.001^a^
	Diarrhea, n (%)	3 (6)	5 (16)	0.25^a^
	Adrenal insufficiency, n (%)	3 (6)	3 (9)	0.67^a^
	Infusion reaction, n (%)	3 (6)	3 (9)	0.67^a^
	Thyroid dysfunction, n (%)	9 (17)	14 (44)	0.017^b^
	Pyrexia, n (%)	5 (10)	5 (15)	0.50^a^
	Rash, n (%)	7 (13)	6 (19)	0.73^b^
CTCAE grade ≥3, n (%)		20 (38)	0 (0)	<0.001^a^
Median duration between initial anti-PD-1 treatment to the first irAE onset, days (range)		40 (0–522)	60 (0–384)	1^c^
ORR, n (%)		24 (46)	21 (66)	0.13^b^
DCR, n (%)		36 (69)	27 (84)	0.19^b^

Differences between groups were identified using ^a^Fisher’s exact test, ^b^chi-square test, or ^c^Student’s t-test. IrAE, immune-related adverse event; CTCAE, Common Terminology Criteria for Adverse Events; PD-1, programmed cell death-1; ORR, overall response rate; DCR, disease control rate.

### Association of Immune Checkpoint Inhibitor Readministration With Clinical Outcomes

In total, 52 patients discontinued anti-PD-1 treatment, and 14 patients were readministered ICIs (**Supplementary Table S2**). All patients had received the same type of anti-PD-1 inhibitor prior to discontinuation. The Kaplan–Meier curves of the 6-week landmark analysis for patients readministered and not readministered anti-PD-1 therapy are shown in **Figure 4**. Two patients who were readministered ICIs after PD were excluded from the PFS analysis. The median PFS was not significantly different between patients with and without the readministration of anti-PD-1 treatment [15.3 (95% CI: 8.3–NE) *vs*. 11.3 (95% CI: 3.5–NE); p = 0.17]. On the other hand, the median OS was significantly longer in patients with ICI readministration than that in patients without ICI readministration [not reached (95% CI: NE–NE) *vs*. not reached (95% CI: 8.4–NE); p = 0.031]. The hazard ratios estimated by the Cox proportional hazards model were as follows: the PFS hazard ratio was 0.46 (95% CI: 0.16–1.41; p = 0.18), and the OS hazard ratio was 0.15 (95% CI: 0.019–1.1; p = 0.063). The characteristics of the initial irAEs stratified by readministration are shown in **Table 5**. The percentage of patients who experienced immune-related pneumonitis at initial ICI treatment was significantly higher in patients who did not receive ICI readministration than that in patients who did receive ICI readministration. The percentage of patients who experienced grade 3 or higher irAEs was not significantly different between patients with ICI readministration and those without ICI readministration. There were no differences between the anti-PD-1 readministration and permanent interruption groups regarding other phenotypes of irAEs or subsequent systemic therapy after anti-PD-1 treatment (**Table 5**). The recurrent and new irAEs that developed after the readministration of anti-PD-1 treatment are detailed in **Table 6**. In 14 patients who were readministered ICIs, two had recurrent irAEs (14%), and two developed new irAEs (14%). Only one patient developed a severe recurrent irAE that was CTCAE grade 3. We have summarized initial irAEs, tumor responses to first anti-PD-1 therapy, and clinical outcomes in patients with continuation and those with or without readministration of anti-PD-1 therapy in **Supplementary Table S3**.

**Table 5:** Characteristics of the initial irAEs and clinical courses, related readministration of anti-PD-1 treatment.

		With readministration of anti-PD-1 treatment(n = 14)	Without readministration of anti-PD-1 treatment(n = 38)	p-value
Phenotypes of IrAEs, n (%)	Pyrexia, n (%)	3 (21)	2 (5)	0.11^a^
	Diarrhea/Colitis, n (%)	2 (14)	1 (3)	0.17^a^
	Adrenal insufficiency, n (%)	2 (14)	1 (3)	0.17^a^
	Liver dysfunction, n (%)	2 (14)	1 (3)	0.17^a^
	Pneumonitis, n (%)	3 (21)	26 (68)	0.004^a^
	Thyroid dysfunction, n (%)	3 (21)	6 (16)	0.69^a^
	Rash, n (%)	2 (14)	5 (13)	1^a^
	Fulminant type 1 diabetes, n (%)	1 (7)	0 (0)	0.27^a^
	Infusion reaction, n (%)	0 (0)	3 (8)	0.56^a^
	Neuropathy, n (%)	0 (0)	2 (5)	1^a^
CTCAE grade ≥3, n (%)		4 (29)	15 (39)	0.53^a^
ORR to the initial anti-PD-1 therapy, n (%)		10 (71)	14 (37)	0.057^b^
Median time from the last administration of the initial anti-PD-1 to the readministration of anti-PD-1, days (range)		70 (22–414)	NA	
Subsequent systemic therapy after anti-PD-1 therapies, n (%)		3 (21)	14 (37)	0.34^a^

Differences between groups were identified using ^a^Fisher’s exact test or ^b^chi-square test. IrAE, immune-related adverse event; CTCAE, Common Terminology Criteria for Adverse Events; ORR, overall response rate; PD-1, programmed cell death-1; NA, not applicable.

**Table 6:** Details of recurrent and new irAEs out of 14 patients with readministered ICI after irAE occurrence.

Case	IrAEs that caused first interruption of ICI	CTCAE grade	IrAEs with readministration	CTCAE grade	ICI continuation	ICI after readministration
1	Pneumonitis	1	Pneumonitis	1	Continued	NA
2	Thyroid dysfunction	3	Thyroid dysfunction	3	Discontinued	Permanently interrupted
3	Liver dysfunction	1	Thyroid dysfunction	1	Continued	NA
4	Diarrhea	2	Anorexia	1	Discontinued	Permanently interrupted

IrAE, immune-related adverse event; CTCAE, Common Terminology Criteria for Adverse Events; ICI, immune checkpoint inhibitor; NA, not applicable.

## Discussion

This study demonstrated the prognostic significance of the occurrence of irAEs, ICI continuation after the development of irAEs, and ICI rechallenge after the interruption of ICIs due to irAEs. Patients with irAEs had a better prognosis than those without irAEs (**Figure 2**), and the continuation or rechallenge of ICIs was associated with better survival times than the permanent interruption of ICIs due to irAEs (**Figures 3**, **4** and **Supplementary Figure S1**). Although some patients with ICI readministration experienced the recurrence of the same or new irAEs, most of these recurrent irAEs were controllable (**Table 6**).

There have been no reports that simultaneously evaluated the significance of ICI continuation and readministration after the occurrence of irAEs in NSCLC patients. In a retrospective study verifying the impact of ICI interruption due to irAEs, the median OS was worse in patients with ICI interruption than that in others with continuous ICI administration (16). Several retrospective studies have examined the safety of ICI rechallenge after initial irAE occurrence in cancer patients and indicated that the safety of ICI rechallenge was acceptable (17–19). A retrospective study of NSCLC demonstrated that among patients with ICI interruption at the time of initial irAE occurrence, ICI rechallenge prolonged OS in patients who had no treatment response before irAE onset (17). While these previous studies focused on only the initial irAEs, our study included all irAEs that occurred during the whole clinical course. The current study showed that among patients with irAEs, although permanent ICI interruption was associated with poor prognosis, ICI readministration and ICI continuation improved prognostic outcomes (**Supplementary Figure S1**). The better prognosis of patients with ICI continuation or readministration than that in patients with permanent ICI interruption may be biologically plausible. The blocking effect of PD-1/PD-L1 is generally expected to diminish with the interruption of ICIs because the binding of the anti-PD-1 antibody to PD-1-positive tumor-infiltrating CD8^+^ T cells is transient (20). Although not related to irAEs, a randomized phase 3b/4 study of NSCLC that compared patients who continued nivolumab for more than 1 year with those who discontinued nivolumab after 1 year of treatment demonstrated that continuous ICI therapy had better clinical outcomes (21). Collectively, these findings suggest the significance of continuously administering ICIs as much as possible after irAE occurrence *via* continuation or readministration.

In the current study, there was no PFS advantage from the continuation or readministration of ICIs in patients with ICI-related irAEs (**Figures 3**, **4**). As a prospective study of patients with nonsquamous NSCLC showed that there was no difference in PFS between nivolumab and docetaxel (1), the efficacy of single-agent ICI therapy might not be able to be evaluated properly by PFS.

Unexpectedly, our study demonstrated no difference in the frequency of irAEs whose CTCAE grade was over 3 between patients with ICI readministration and those with permanent ICI interruption (**Tables 4**, **5**). This result might suggest that clinicians aggressively readministered ICIs to patients whose irAEs had been severe but improved. Indeed, better survival outcomes were observed in patients who had experienced grade 3–4 irAEs and received the readministration of anti-PD-1 therapy (**Supplementary Figure S2**). Although Johnson et al. (22) suggested that severe or life-threatening toxicity is one of the factors that argues against ICI rechallenge, the readministration of ICIs might be considered in patients whose irAEs had been severe but recovered. However, it is noteworthy in the current study that the frequency of pneumonitis as an irAE was significantly higher in patients who discontinued ICIs and in those who permanently interrupted ICIs (**Tables 4**, **5**). In addition, our study suggests that the readministration of anti-PD-1 therapy had no survival benefit in patients with pneumonitis (**Supplementary Figure S2**). Several meta-analyses have reported that pneumonitis is one of the most common fatal irAEs in patients treated with ICIs (23, 24). Therefore, although there has been no evidence that the continuous administration or readministration of ICIs tends to lead to fatal irAEs, it should be noted that the continuation or readministration of ICIs to patients who experienced irAEs such as pneumonitis, which could be fatal if exacerbated, should be carefully determined on a patient-by-patient basis.

The limitations of the current study include the relatively small number of patients with ICI readministration and the retrospective nature of the study. Clinicians might have tended to avoid continuing and readministering ICIs to patients with irAEs such as pneumonitis, which could be fatal if exacerbated. There is no detailed analysis for each irAE in this study. In addition, response rate to initial anti-PD-1 therapies in patients with readministration tended to be higher than that in patients without readministration (ORR 71% *vs*. 37%, p = 0.057; **Table 5**). There is a possibility that clinicians might have tended to readminister anti-PD-1 therapy to patients with good tumor response to initial ICI even with irAEs.

In summary, we retrospectively investigated the clinical significance of the continuation and readministration of single-agent anti-PD-1 therapy in NSCLC patients with ICI-related irAEs. The continuation and readministration of ICIs significantly prolonged OS, and their safety was acceptable. A future prospective study is needed to establish optimal treatment strategies for patients with irAEs.

## Data Availability Statement

The raw data supporting the conclusions of this article will be made available by the authors, without undue reservation.

## Ethics Statement

The studies involving human participants were reviewed and approved by Niigata University Graduate School of Medical and Dental Sciences, Niigata Prefectural Shibata Hospital, Niigata City General Hospital, Niigata Cancer Center Hospital, Saiseikai Niigata Hospital, Nishi-Niigata Chuo National Hospital, Niigata Prefectural Central Hospital, Nagaoka Chuo General Hospital, Nagaoka Red Cross Hospital, Niigata Medical Center. The patients/participants provided their written informed consent to participate in this study.

## Author Contributions

TO devised this study. TO and SW designed the protocol. SW, TO, SS, KN, KI, RK, TM, TA, SM, HT, MO, MTe, NM, TI, AI, KS, HY, MH enrolled patients into the study. TF and SW co-wrote this manuscript, conducted data analysis, and prepared figures and tables. All authors contributed to the article and approved the submitted version.

## Funding

The primary author was supported by a grant from the Niigata Medical Association, Japan.

## Conflict of Interest

SW reports grants and personal fees from AstraZeneca, personal fees from Chugai Pharma, personal fees from Ono Pharmaceutical, personal fees from Bristol-Myers, grants and personal fees from Boehringer Ingelheim, personal fees from Eli Lilly, personal fees from MSD, personal fees from Taiho Pharmaceutical, personal fees from Pfizer, personal fees from Novartis, and personal fees from Daiichi Sankyo outside the submitted work. TO reports personal fees from Boehringer Ingelheim, personal fees from MSD, personal fees from Eli Lilly, personal fees from AstraZeneca, personal fees from Chugai-pharm, and personal fees from Bristol-Myers Squibb outside the submitted work. SS reports personal fees from AstraZeneca, personal fees from Chugai Pharma, personal fees from Taiho Pharmaceutical, and personal fees from MSD outside the submitted work. KN reports personal fees from AstraZeneca, personal fees from Boehringer Ingelheim, personal fees from Taiho Pharmaceutical, and personal fees from MSD outside the submitted work. AI reports personal fees from AstraZeneca, personal fees from Chugai Pharma, personal fees from Bristol-Myers, personal fees from Boehringer Ingelheim, personal fees from Ono Pharmaceutical, personal fees from Taiho Pharmaceutical, personal fees from Novartis International AG, and personal fees from Daiichi Sankyo Company outside the submitted work. SH reports personal fees from GlaxoSmithKline Inc. outside the submitted work. TA reports personal fees from Eli Lilly Japan, personal fees from Chugai Pharmaceutical, personal fees from Taiho Pharmaceutical, personal fees from Ono Pharmaceutical, personal fees from Bristol-Myers Squibb, personal fees from AstraZeneca, and personal fees from Mylan outside the submitted work. SM reports personal fees from Chugai Pharmaceutical, personal fees from ONO Pharmaceutical, personal fees from AstraZeneca, personal fees from Eli Lilly, personal fees from MSD, personal fees from Boehringer Ingelheim, personal fees from Taiho Pharmaceutical, personal fees from Novartis, personal fees from Bristol-Myers Squibb, and personal fees from Kyowa Hakko Kirin outside the submitted work. HT reports grants and personal fees from Bristol-Myers Squibb, grants and personal fees from Eli Lilly, grants and personal fees from MSD, grants and personal fees from Taiho pharmaceutical, grants and personal fees from Pfizer, grants and personal fees from Novartis, grants and personal fees from Chugai pharmaceutical, grants and personal fees from Astra Zeneca, grants and personal fees from Boehringer Ingelheim, grants and personal fees from Ono pharmaceutical, and grants and personal fees from Merck outside the submitted work. MO reports personal fees from AstraZeneca, personal fees from Ono Pharmaceutical, personal fees from Bristol-Myers, personal fees from Boehringer Ingelheim, personal fees from MSD, and personal fees from Taiho Pharmaceutical outside the submitted work. NA reports personal fees from Meiji Seika Pharma, grants from MSD, and personal fees from MSD outside the submitted work. MH reports personal fees from Boehringer-Ingelheim Japan, personal fees from AstraZeneca, personal fees from Taiho Pharmaceutical, and personal fees from Daiichi Sankyo outside the submitted work. YO reports personal fees from Boehringer-Ingelheim Japan and personal fees from Meiji Seika Pharma outside the submitted work. TKo reports personal fees from AstraZeneca, Boehringer-Ingelheim, Sanofi Genzyme, Novartis, Daiichi Sankyo, Kyorin Pharmaceutical, and GlaxoSmithKline outside the submitted work. TKi reports grants and personal fees from Chugai Pharma, grants and personal fees from Boehringer Ingelheim, grants and personal fees from Eli Lilly, grants and personal fees from MSD K.K., personal fees from Astellas Pharma Inc., grants and personal fees from Taiho Pharmaceutical Co., Ltd., personal fees from Bristol-Myers Squibb Company, personal fees from Pfizer Japan Inc., grants and personal fees from Daiichi Sankyo Co., Ltd., personal fees from Taisho Toyama Pharmaceutical Co., Ltd., personal fees from Janssen Pharmaceutical K.K., personal fees from Japan BCG Laboratory, grants and personal fees from Ono Pharmaceutical Co., Ltd., personal fees from Novartis Pharma K.K., personal fees from Mylan N.V., grants and personal fees from AstraZeneca, personal fees from Roche Diagnostics K.K., grants and personal fees from Shionogi & Co., Ltd., grants from TEIJIN PHARMA Ltd., and grants from KYORIN Pharmaceutical Co., Ltd., outside the submitted work.

The remaining authors declare that the research was conducted in the absence of any commercial or financial relationships that could be construed as a potential conflict of interest.

## Publisher’s Note

All claims expressed in this article are solely those of the authors and do not necessarily represent those of their affiliated organizations, or those of the publisher, the editors and the reviewers. Any product that may be evaluated in this article, or claim that may be made by its manufacturer, is not guaranteed or endorsed by the publisher.

## Abbreviations

ICI, immune checkpoint inhibitor; PD-1, programmed cell death-1; PD-L1, programmed cell death ligand-1; NSCLC, non-small-cell lung cancer; ILD, interstitial lung disease; GGO, ground-glass opacity; PFS, progression-free survival; OS, overall survival; irAE, immune-related adverse event; NOS, not otherwise specified; CR, complete response; PR, partial response; SD, stable disease; PD, progressive disease; ORR, objective response rate; DCR, disease control rate.
