# Supplementary material for: The Prognostic Significance of the Continuous Administration of Anti-PD-1 Antibody via Continuation or Rechallenge After the Occurrence of Immune-Related Adverse Events
Source: Front Oncol. 2021 Sep 24;11:704475. doi: 10.3389/fonc.2021.704475 (PMC8498597; doi:10.3389/fonc.2021.704475)
Supplement: Supplementary file 1 [file Presentation_1.pptx]

## Slide 1
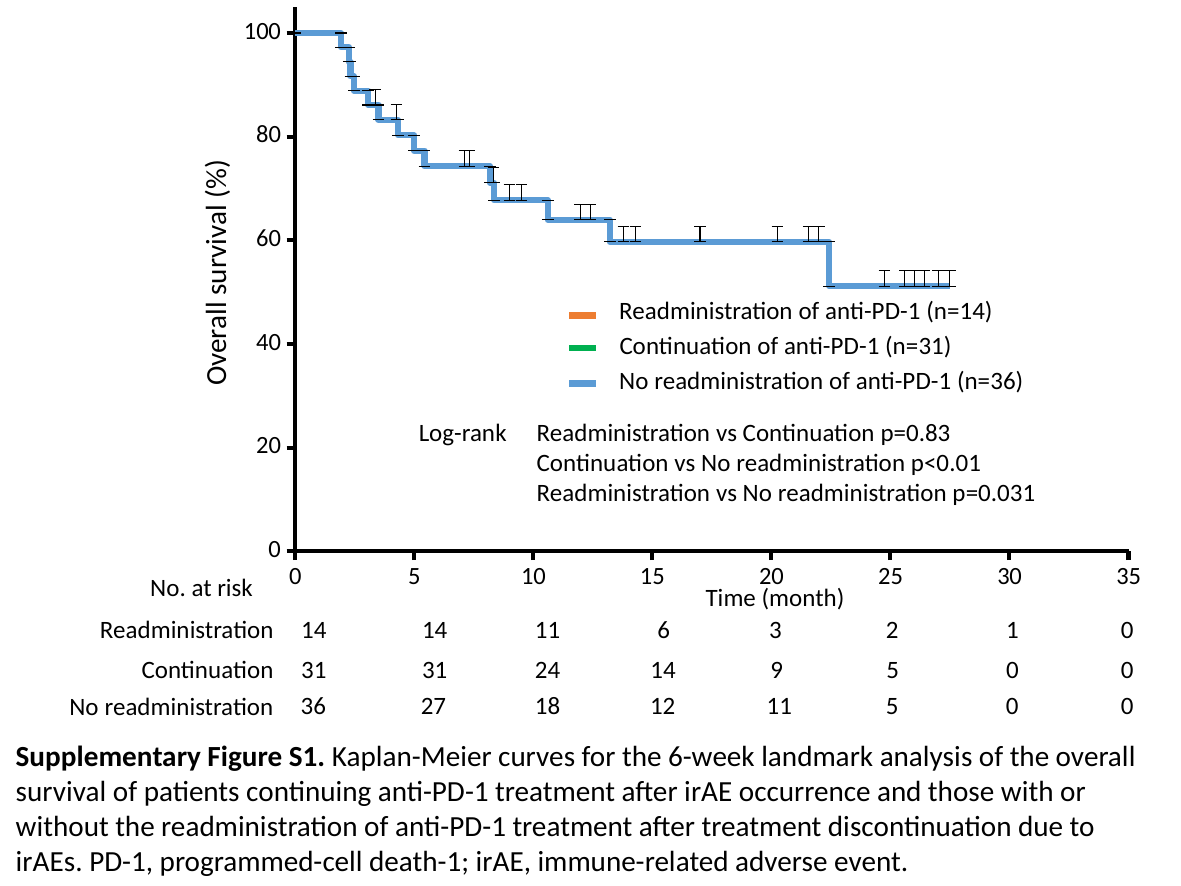

### Chart
| Category | | | |
|---|---|---|---|Overall survival (%)
Readministration of anti-PD-1 (n=14)
Continuation of anti-PD-1 (n=31)
No readministration of anti-PD-1 (n=36)
Log-rank	Readministration vs Continuation p=0.83
 	Continuation vs No readministration p<0.01
 	Readministration vs No readministration p=0.031
No. at risk
Time (month)
Readministration
14
14
11
6
3
2
1
0
Continuation
31
31
24
14
9
5
0
0
36
27
18
12
11
5
0
0
No readministration
Supplementary Figure S1. Kaplan-Meier curves for the 6-week landmark analysis of the overall survival of patients continuing anti-PD-1 treatment after irAE occurrence and those with or without the readministration of anti-PD-1 treatment after treatment discontinuation due to irAEs. PD-1, programmed-cell death-1; irAE, immune-related adverse event.
