# Supplementary material for: The Prognostic Significance of the Continuous Administration of Anti-PD-1 Antibody via Continuation or Rechallenge After the Occurrence of Immune-Related Adverse Events
Source: Front Oncol. 2021 Sep 24;11:704475. doi: 10.3389/fonc.2021.704475 (PMC8498597; doi:10.3389/fonc.2021.704475)
Supplement: Supplementary file 2 [file Presentation_2.pptx]

## Slide 1
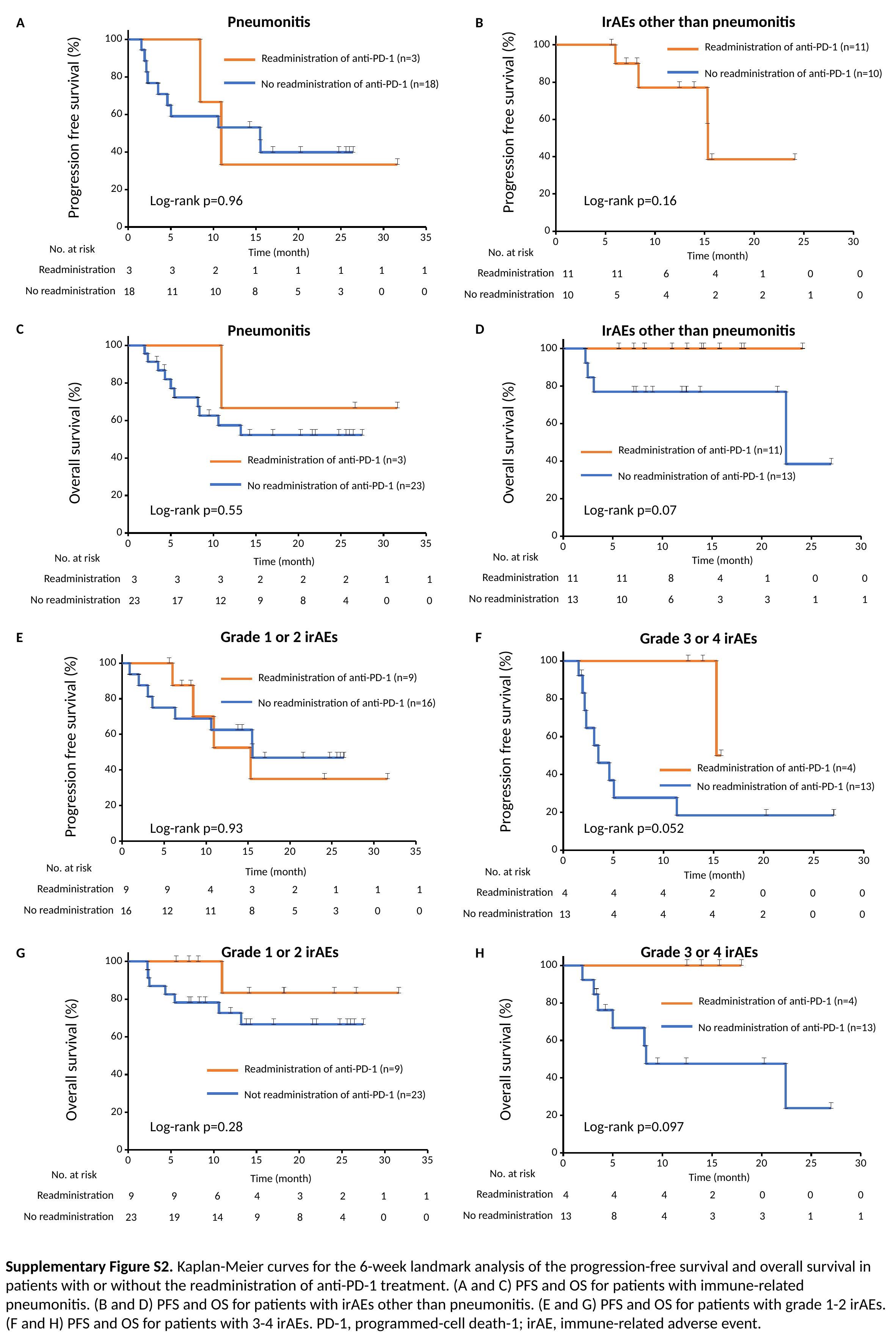

Pneumonitis
IrAEs other than pneumonitis
A
B
### Chart
| Category | | |
|---|---|---|
### Chart
| Category | | |
|---|---|---|Readministration of anti-PD-1 (n=11)
Readministration of anti-PD-1 (n=3)
No readministration of anti-PD-1 (n=10)
No readministration of anti-PD-1 (n=18)
Progression free survival (%)
Progression free survival (%)
Log-rank p=0.96
Log-rank p=0.16
No. at risk
No. at risk
Time (month)
Time (month)
Readministration
1
3
3
1
1
2
1
1
Readministration
11
11
1
0
6
4
0
No readministration
0
18
11
5
0
10
8
3
No readministration
10
5
2
0
4
2
1
C
Pneumonitis
D
IrAEs other than pneumonitis
### Chart
| Category | | |
|---|---|---|
### Chart
| Category | | |
|---|---|---|Overall survival (%)
Overall survival (%)
Readministration of anti-PD-1 (n=11)
Readministration of anti-PD-1 (n=3)
No readministration of anti-PD-1 (n=13)
No readministration of anti-PD-1 (n=23)
Log-rank p=0.55
Log-rank p=0.07
No. at risk
No. at risk
Time (month)
Time (month)
Readministration
11
11
1
0
8
4
0
Readministration
1
3
3
2
1
3
2
2
No readministration
13
10
3
1
6
3
1
No readministration
0
23
17
8
0
12
9
4
Grade 1 or 2 irAEs
E
F
Grade 3 or 4 irAEs
### Chart
| Category | | |
|---|---|---|
### Chart
| Category | | |
|---|---|---|Readministration of anti-PD-1 (n=9)
No readministration of anti-PD-1 (n=16)
Progression free survival (%)
Progression free survival (%)
Readministration of anti-PD-1 (n=4)
No readministration of anti-PD-1 (n=13)
Log-rank p=0.93
Log-rank p=0.052
No. at risk
No. at risk
Time (month)
Time (month)
Readministration
1
9
9
2
1
4
3
1
Readministration
4
4
0
0
4
2
0
No readministration
0
16
12
5
0
11
8
3
No readministration
13
4
2
0
4
4
0
Grade 1 or 2 irAEs
Grade 3 or 4 irAEs
G
H
### Chart
| Category | | |
|---|---|---|
### Chart
| Category | | |
|---|---|---|Readministration of anti-PD-1 (n=4)
No readministration of anti-PD-1 (n=13)
Overall survival (%)
Overall survival (%)
Readministration of anti-PD-1 (n=9)
Not readministration of anti-PD-1 (n=23)
Log-rank p=0.28
Log-rank p=0.097
No. at risk
No. at risk
Time (month)
Time (month)
Readministration
4
4
0
0
4
2
0
Readministration
1
9
9
3
1
6
4
2
No readministration
13
8
3
1
4
3
1
No readministration
0
23
19
8
0
14
9
4
Supplementary Figure S2. Kaplan-Meier curves for the 6-week landmark analysis of the progression-free survival and overall survival in patients with or without the readministration of anti-PD-1 treatment. (A and C) PFS and OS for patients with immune-related pneumonitis. (B and D) PFS and OS for patients with irAEs other than pneumonitis. (E and G) PFS and OS for patients with grade 1-2 irAEs. (F and H) PFS and OS for patients with 3-4 irAEs. PD-1, programmed-cell death-1; irAE, immune-related adverse event.
