# Supplementary material for: The Prognostic Significance of the Continuous Administration of Anti-PD-1 Antibody via Continuation or Rechallenge After the Occurrence of Immune-Related Adverse Events
Source: Front Oncol. 2021 Sep 24;11:704475. doi: 10.3389/fonc.2021.704475 (PMC8498597; doi:10.3389/fonc.2021.704475)
Supplement: Supplementary file 3 [file Table_1.docx]

| **Supplementary Table 1. Patients’ characteristics at initial anti-PD-1 therapy, with or without anti-PD-1 interruption due to irAE.** | | | | |
| --- | --- | --- | --- | --- |
| Clinical features | | Anti-PD-1 treatment interruption (total, n=52) | Anti-PD-1 treatment continuation (total, n=32) | *P* value |
| Median age, years (range) |  | 67 (41-83) | 68 (45-84) | 0.95^a^ |
| Sex, n (%) | Female/male | 8 (15) /44 (85) | 4 (13) /28 (88) | 1^c^ |
| Smoking status, n (%) | Current or former | 45 (87) | 31 (97) | 0.15^c^ |
|  | Never | 7 (13) | 1 (3) |  |
| PS, n (%) | 0-1 | 45 (87) | 29 (91) | 0.73^c^ |
|  | ≥2 | 7 (13) | 3 (9) |  |
| Stage, n (%) | III | 7 (13) | 5 (16) | 0.42^b^ |
|  | IV | 27 (52) | 12 (38) |  |
|  | Recurrent | 18 (35) | 15 (47) |  |
| Histology, n (%) | Adenocarcinoma | 19 (37) | 11 (34) | 0.86^b^ |
|  | Squamous cell carcinoma | 27 (52) | 16 (50) |  |
|  | Others | 6 (12) | 5 (16) |  |
| Driver mutation, n (%) | *EGFR* | 1 (1) | 0 (0) | 1^c^ |
| Treatment line of anti-PD-1  therapy, n (%) | 1^st^ line | 14 (27) | 6 (19) | 0.39^b^ |
|  | 2^nd^, 3^rd^ line | 38 (73) | 26 (81) |  |
| PD-L1 expression, n (%) | ≥50% | 19 (37) | 7 (22) | 0.31^b^ |
|  | 1-49% | 3 (6) | 2 (6) |  |
|  | <1% | 2 (4) | 4 (13) |  |
|  | Unknown | 28 (54) | 19 (59) |  |
| Anti-PD-1 therapy, n (%) | Nivolumab | 33 (63) | 23 (72) | 0.43^b^ |
|  | Pembrolizumab | 19 (37) | 9 (28) |  |
| Differences between groups were identified using ^a^Student's t-test, ^b^Chi-Square test or ^c^Fisher’s exact test. IrAE, immune-related adverse event; PS, performance status; PD-1, programmed-cell death-1; PD-L1, PD-ligand 1; EGFR, epidermal-growth factor receptor; irAE, immune-related adverse event. | | | | |
