# Supplementary material for: The Prognostic Significance of the Continuous Administration of Anti-PD-1 Antibody via Continuation or Rechallenge After the Occurrence of Immune-Related Adverse Events
Source: Front Oncol. 2021 Sep 24;11:704475. doi: 10.3389/fonc.2021.704475 (PMC8498597; doi:10.3389/fonc.2021.704475)
Supplement: Supplementary file 4 [file Table_2.docx]

| **Supplementary Table 2. Patients' characteristics at initial anti-PD-1 therapy, with or without readministration of anti-PD-1 treatment.** | | | | |
| --- | --- | --- | --- | --- |
| Clinical features | | Readministration of anti-PD-1 treatment (total, n=14) | No readministration of anti-PD-1 treatment (total, n=38) | *p* Value |
| Median age, years (range) |  | 64 (41-83) | 67 (45-82) | 0.24^a^ |
| Sex, n (%) | Female/male | 2 (14)/12 (86) | 6 (16)/32 (84) | 1^c^ |
| Smoking status, n (%) | Current or former | 12 (86) | 33 (87) | 1^c^ |
|  | Never | 2 (14) | 5 (13) |  |
| PS, n (%) | 0-1 | 13 (93) | 4 (84) | 0.66^c^ |
|  | ≥ 2 | 1 (7) | 20 (16) |  |
| Stage, n (%) | III | 3 (21) | 4 (11) | 0.57^b^ |
|  | IV | 7 (50) | 20 (53) |  |
|  | Recurrent | 4 (29) | 14 (37) |  |
| Histology, n (%) | Adenocarcinoma | 4 (29) | 15 (39) | 0.54^b^ |
|  | Squamous cell carcinoma | 9 (64) | 18 (47) |  |
|  | Others | 1 (7) | 5 (13) |  |
| Driver mutation, n (%) | *EGFR* | 1 (1) | 0 (0) | 1^c^ |
| Treatment line of anti-PD-1  therapy, n (%) | 1^st^ line | 5 (36) | 9 (24) | 0.48^c^ |
|  | 2^nd^, 3^rd^ line | 9 (64) | 29 (76) |  |
| PD-L1 expression, n (%) | ≥50% | 5 (36) | 14 (37) | 0.66^b^ |
|  | 1-49% | 1 (7) | 2 (5) |  |
|  | <1% | 1 (7) | 1 (3) |  |
|  | Unknown | 7 (50) | 21 (55) |  |
| Anti-PD-1 therapy, n (%) | Nivolumab | 9 (64) | 24 (63) | 0.43^c^ |
|  | Pembrolizumab | 5 (36) | 14 (37) |  |
| Differences between groups were identified using ^a^Student's t-test, ^b^Chi-Square test or ^c^Fisher’s exact test. PS, performance status; PD-1, programmed-cell death-1; PD-L1, PD-ligand 1; irAE, immune-related adverse event; EGFR, epidermal-growth factor receptor. | | | | |
