# Supplementary material for: The Prognostic Significance of the Continuous Administration of Anti-PD-1 Antibody via Continuation or Rechallenge After the Occurrence of Immune-Related Adverse Events
Source: Front Oncol. 2021 Sep 24;11:704475. doi: 10.3389/fonc.2021.704475 (PMC8498597; doi:10.3389/fonc.2021.704475)
Supplement: Supplementary file 5 [file Table_3.docx]

| **Supplementary table 3. IrAEs, tumor responses and outcomes in subgroup.** | | | | | |
| --- | --- | --- | --- | --- | --- |
|  |  | Continuation of  anti-PD-1 treatment  (n=32) | Readministration of anti- PD-1 treatment (n=14) | No readministration of anti- PD-1 treatment (n=38) | *P* value |
| Phenotype of initial irAEs, n (%) | Pyrexia, n (%) | 5 (15) | 3 (21) | 2 (5) | 0.16^a^ |
|  | Diarrhea/colitis, n (%) | 5 (16) | 2 (14) | 1 (3) | 0.11^a^ |
|  | Adrenal insufficiency, n (%) | 3 (9) | 2 (14) | 1 (3) | 0.21^a^ |
|  | Liver dysfunction, n (%) | 0 (0) | 2 (14) | 1 (3) | 0.071^a^ |
|  | Pneumonitis, n (%) | 1 (3) | 3 (21) | 26 (68) | <0.001^a^ |
|  | Thyroid dysfunction, n (%) | 14 (44) | 3 (21) | 6 (16) | 0.04^a^ |
|  | Rash, n (%) | 6 (19) | 2 (14) | 5 (13) | 0.92^a^ |
|  | Fulminant type 1 diabetes, n (%) | 0 (0) | 1 (7) | 0 (0) | 0.17^a^ |
|  | Infusion reaction, n (%) | 3 (9) | 0 (0) | 3 (8) | 0.74^a^ |
|  | Neuropathy, n (%) | 0 (0) | 0 (0) | 2 (5) | 0.65^a^ |
| CTCAE Grade ≥3, n (%) | | 0 (0) | 4 (29) | 15 (39) | <0.001^a^ |
| Median duration between initial anti-PD-1 treatment to the 1^st^ irAE onset, days (range) | | 60 (0-384) | 85  (1-522) | 28  (0-332) | 0.15^c^ |
| ORR, n (%) | | 21 (66) | 10 (71) | 14 (37) | 0.02^b^ |
| DCR, n (%) | | 27 (84) | 14 (100) | 22 (58) | 0.002^b^ |
| 6-week landmark analysis of median PFS,  month (95% CI) | | 15.4 (9-NE) | 15.3 (8.3-NE) | 11.3 (3.5-NE) | 0.59^d^ |
| 6-week landmark analysis of median OS, month (95% CI) | | Not reached (NE-NE) | Not reached (NE-NE) | not reached (8.4-NE) | 0.025^d^ |
| Median follow-up, days (range) | | 411 (28-830) | 393 (157-886) | 259 (27-770) | 0.16^c^ |
| Differences between groups were identified using ^a^Fisher’s exact test, ^b^Chi-Square test, ^c^one-way ANOVA, ^d^Log-rank test. CTCAE, Common Terminology Criteria for Adverse Events; PD-1, programmed-cell death-1; irAE, immune-related adverse event; ORR, objective response rate; DCR, disease control rate; PFS, progression-free survival; OS, overall survival; NE, not evaluable. | | | | | |
